# Walking Backward to Ensure Risk Management of Large Language Models in Medicine

**DOI:** 10.1017/jme.2025.10132

**Published:** 2025-01-01

**Authors:** Daria Onitiu, Sandra Wachter, Brent Mittelstadt

**Affiliations:** 1 Oxford Internet Institute, University of Oxford, United Kingdom; 2 Hasso Plattner Institute, Potsdam, Germany

**Keywords:** Medical Device Regulation, Large Language Models (LLMs), Risk Management, EU law, Medical device safety, Artificial Intelligence (AI)

## Abstract

This paper examines in what way providers of specialized Large Language Models (LLM) pre-trained and/or fine-tuned on medical data, conduct risk management, define, estimate, mitigate and monitor safety risks under the EU Medical Device Regulation (MDR). Using the example of an Artificial Intelligence (AI)-based medical device for lung cancer detection, we review the current risk management process in the MDR entailing a “forward-walking” approach for providers articulating the medical device’s clear intended use, and moving on sequentially along the definition, mitigation, and monitoring of risks. We note that the forward-walking approach clashes with the MDR requirement for articulating an intended use, as well as circumvents providers reasoning around the risks of specialised LLMs. The forward-walking approach inadvertently introduces different intended users, new hazards for risk control and use cases, producing unclear and incomplete risk management for the safety of LLMs. Our contribution is that the MDR risk management framework requires a backward-walking logic. This concept, similar to the notion of “backward-reasoning” in computer science, entails sub-goals for providers to examine a system’s intended user(s), risks of new hazards and different use cases and then reason around the task-specific options, inherent risks at scale and trade-offs for risk management.

## Introduction

This paper is concerned with the regulation of large language models (LLMs) in medicine under the EU Medical Device frame-work:^1^ how can providers use a pre-trained and general-purpose system, fine-tuned to a specific medical task, and make claims about their safety and performance when deployed for clinical decision-support?^2^ LLMs are current advancements in artificial intelligence (AI) which are intended to generate human-like text and which can be repurposed and adapted to a range of different domains and tasks.^3^ Our work contributes to discussions on the novel risks and regulatory challenges of these advanced AI systems in EU policy and academic scholarship, which situate issues to demonstrate claims regarding the safety, performance and effectiveness of LLMs when these models are adapted to a medical purpose.^4^ The aim of this paper is to review these concerns, while making tensions for providers to *reason around* the risks of LLMs apparent. Focusing on the regulatory tensions in the EU Medical Device Regulation (MDR)^5^ — one of two regulatory frameworks applicable to the certification of LLMs as medical device^6^ — we specifically focus on the MDR’s risk management framework for the provider to define, estimate, mitigate and monitor performance and safety risks and how the general capabilities of these specialized models contravene the well-established risk management approach in the MDR.

Risk management is the backbone for manufacturers and providers of AI-based medical devices to demonstrate their safety and performance, as well as clinical benefit under the MDR.^7^ This risk management process also covers software, hardware and components entailing AI techniques, such as machine learning and deep learning techniques intended to assist in the detection of lung cancer in X-rays. Using an example of an AI lung cancer detection tool, we examine how providers need to identify and mitigate associated risks, such as measurement errors or automation bias, and evaluate these problems within the system’s intended use, as well as establish post-monitoring of the device’s safety and performance across its lifecycle.^8^ Without a robust risk management plan, claims about the system’s intended use and risks will be unclear or underspecified, thereby endangering patient safety.

Providers of medical LLMs are required to adopt this sequential approach to risk management under the MDR; that is, to define the intended use and use this forward-walking logic along estimation, mitigation, and monitoring of risks. Medical LLMs, such as the specialized models fine-tuned in the medical domain, evaluated, and locked down through instruction tuning, prompt engineering and reinforcement learning from human feedback (RLHF) are positioned to challenge these well-defined risk management principles. The nature of a general-purpose system, allowing providers to optimize a model for a medical related task — for example, medical question-answering on the presence of lung cancer — preclude normative propositions around the device’s intended use. Instead, a forward-walking logic may produce incomplete specifications about the stages of risk management, undermining the robust and overall appreciation concerning the safety and performance of medical LLMs.

Specifically, fine-tuning and optimizing medical LLMs may produce three different areas of concern for providers conducting effective risk management. First, dynamic specifications around the medical LLMs task-specific options need to be defined by virtue of the intended use(r)’s and interactions with the model. Second, hazards and sources of potential harm cannot be estimated based on the LLM’s intended use but require the provider to focus on trade-offs arising from fine-tuning. Finally, the provider’s articulation of the intended use needs to be far more open-ended to judge certain specific risks at scale — including hallucinations where the model produces fabricated output including incorrect information — which must be monitored after the system’s deployment.

To include these new specifications — task-specific options, trade-offs, and inherent risks at scale — for the certification of medical LLMs under the MDR requires a revision of the logic underpinning risk management. Providers of medical LLMs need to walk “backward” to identify and evaluate a model’s intended use under the MDR. This entails a combination of hypothesis-driven work and the sub-goals, task-specific options, trade-offs, and inherent risks for the provider to demonstrate the system’s intended use. Using this approach will ensure that medical LLMs can be subject to risk management under the MDR.

### Defining the “Forward-Walking” Logic

The following sections will introduce the main logic underpinning risk management under the MDR, and how performance and safety assurances regarding specialized, medical LLMs fit into this frame-work. To elaborate on this, we first need to clarify three elements — the identification, risk estimation, mitigation, and monitoring — under the MDR.^9^

**Figure 1:**
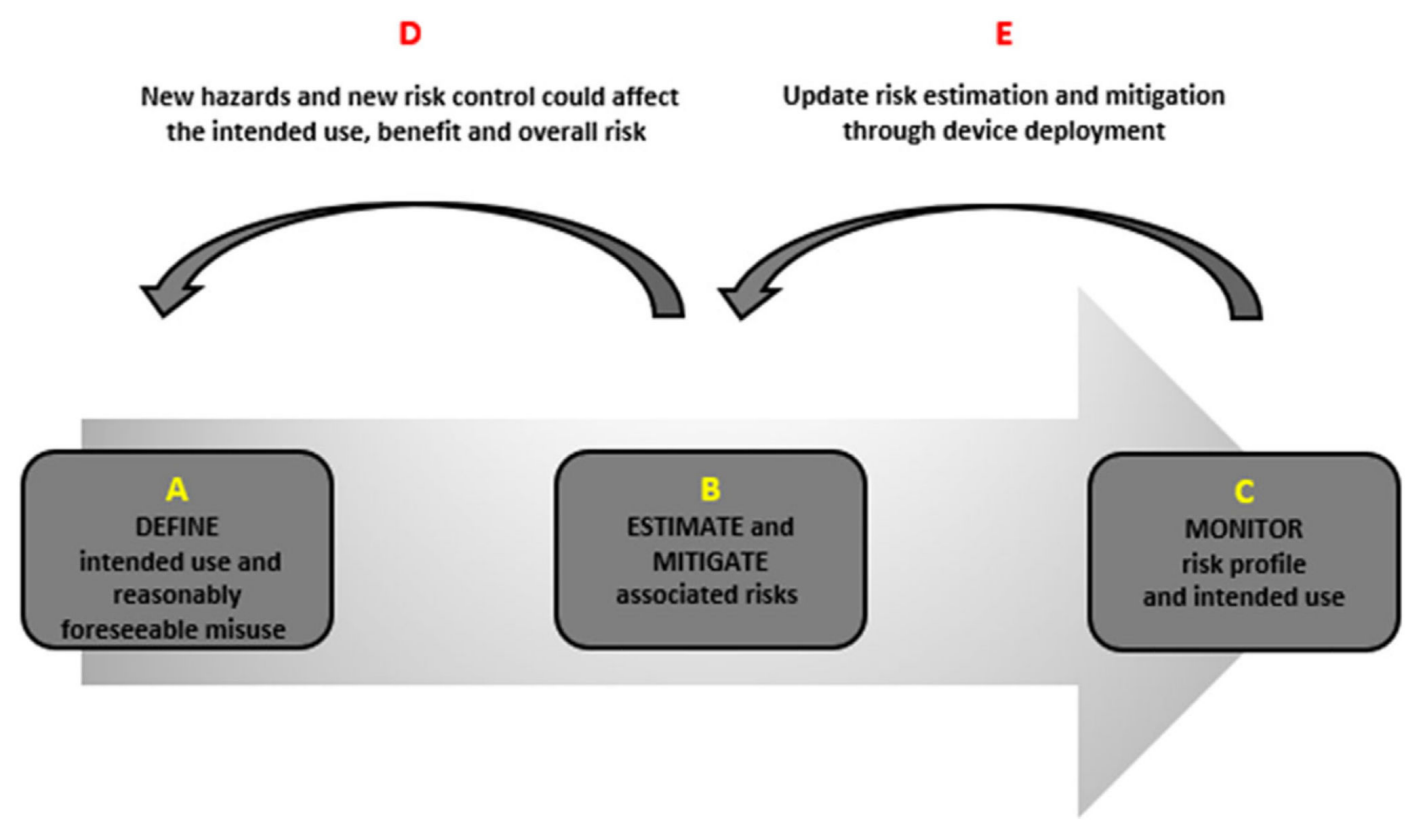
An illustration of the risk management lifecycle is based on the manufacturer’s articulation of the intended purpose and use in the MDR. We describe this approach as “forward-walking” to emphasize that risk assessment stems from a clearly articulated intended use and progresses through the stages of risk management to ensure the safety and performance of the device. These stages reinforce each other, constituting an iterative process and a feedback loop for ensuring patient safety.

By way of illustration, imagine a provider who wants to deploy a new AI-based medical device to automate the analysis of X-rays and assist with the detection of lung cancer. To conduct risk management would require the provider to construct an “iterative process.”^10^ This may entail the operational implications and limits of the device, the context in which the device is intended to be used, who can use this system and for what clinical conditions, amongst other factors.^11^ In addition, some risks — for example, measurement errors and usability risks — require providers to adopt certain safeguards.^12^ This may range from design specifications to instruction for use, such as data quality requirements or a warning system for ensuring human oversight.^13^ Finally, providers need to monitor the system’s intended use, associated risks, and effectiveness of risk control when deployed on the ground.^14^ Figure 1 and the proceeding discussion simplify the stages of risk management, considering this illustrative example.

#### Define the Intended Use and Reasonably Foreseeable Misuse

Providers need to articulate potential hazards of our lung cancer detection software. This involves specifying the “intended use” and “reasonably foreseeable misuse,” incorporating specific properties for effective risk management.^15^ Following this thought process, the manufacturer could specify information about the different stages and types of lung cancer, indications for the system’s use in cancer care, operating principles for the system to discriminate between different stages of lung cancer in a medical image, the type of user profile and training required for assisting clinical decision-making, the type of environment used in emergency care, and the subpopulations for which the system has been tested and evaluated.^16^

The definition of the intended use and reasonably foreseeable misuse is important as it effectively informs the next and sequential steps in the risk management cycle. For example, if the manufacturer does not identify details about the AI cancer detection performance and its functionality, as well as risks of misuse of the device, then any subsequent risk analysis would likely produce an overestimation or underestimation of risk (Figure 1, Point B). Moreover, and particularly in relation to novel technologies, post-production activities to monitor and respond to any emergent risks are also important features that help providers to respond to adverse events and new instances of misuse^17^ (Figure 1, Point C).

#### Estimate and Mitigate Risks Associated with the Intended Use

Above, we elaborated on why the definition of the intended use is important when looking holistically at the risk management cycle. Turning to the estimation and mitigation of the system’s risks, this stage allows the manufacturer of the AI lung cancer detection software to pinpoint so-called “hazards and hazardous situations” and estimate the associated risks, based on “probability and severity of occurrence of harm.”^18^ This could entail the estimation of a range of risks — for instance, measurement errors and risks of over-reliance — that could lead to a “sequence of events” causing false diagnosis and patient harm.^19^ Subsequently, and based on a “risk acceptability policy,” the developer may implement and assess “risk controls.”^20^ For instance, data quality controls and instructions for use can limit the performance and usability risks to an acceptable level when evaluated and validated. In this respect, the provider would have a risk policy in place. Using this risk policy after risk mitigation, the manufacturer decides on the overall risk to judge whether all risks have been reduced “as far as possible”^21^ and to an acceptable degree.^22^ A unique risk of our AI lung cancer detection software is that any new interactions, such as learning based on new and real-world data and interaction with the intended users (Figure 1, Point E), or through bias mitigation (Figure 1, Point D), may introduce new risks of bias and fairness.^23^ The manufacturer thereby needs to conduct iterative evaluations of risk and control to ensure that device performance remains “suitable for its intended use” without adversely undermining that risk policy.^24^

#### Monitor the Risk Profile and Intended Use

Hence, the device’s risk policy including the risk profile initiates an iterative process for the developer to uphold the system’s intended use and reasonably foreseeable misuse throughout its lifecycle. For our AI lung cancer detection software, the developer needs not only to ensure that the device is performing as intended, but that the propositions about risks and evidence-based conclusions (the so-called “benefit-risk determination”), such as its indications for use in cancer care, remain valid throughout the system’s lifecycle.^25^ In this regard, “postproduction activities and data” enable updates to the risk management files and adjustments to risk estimates and controls after the system’s deployment.^26^ By way of illustration, postproduction data may reveal emergent risks about the AI cancer detection software, such as the system learning to detect different stages of cancer for new subpopulations, or other issues, such as new problems of automation bias arising from risk control.^27^ Moreover, risks of misuse, particularly “off-label” use, form part of “post-market surveillance” and the monitoring framework.^28^ If these instances happen, then the developer needs to investigate whether the device’s intended use still meets the risk acceptability criteria and adopt risk control measures.^29^

Following this description of the risk management framework, its logic underpins a sequential and iterative process to ensure the performance and safety of a medical device. This means that manufacturers of AI-based medical devices need to have a plan and system in place to manage risks — like the measurement error and usability concerns in our example — throughout the lifecycle. Moreover, manufacturers need to remain responsive to change in the overall risk policy.

In doing so, the manufacturer needs to assure: “Am I building the right thing for task X and does this tool perform as intended for this specific task in an intended development setting and throughout deployment?” Notably, this framework directs manufacturers to reason around the risks of medical devices in a specific way, using the intended use as a starting point and then moving forward to the stages of risk estimation, control, and monitoring sequentially.

### Does Forward-Walking Work with Medical Large Language Models?

The second part regarding the question of how performance and safety assurances regarding specialized, medical LLMs fit into this MDR framework is descriptive and normative. It is descriptive because it asks, “How do providers of medical LLMs demonstrate that the sequential approach has been followed?” As noted above, risk management is also a framework for manufacturers to reason about the risks of AI-based medical devices. Therefore, whether current design and fine-tuning of medical LLMs disturb propositions about the device’s intended use is a normative question that needs to be clarified. Both lenses — the descriptive and evaluative — are needed for ensuring that medical LLMs fulfill standards of safety and performance and do not endanger patient safety.

Medical LLMs are positioned to challenge the MDR risk management framework. We choose the term “medical LLMs” to describe specialized LLMs that have been pre-trained and/or fine-tuned on medical data.^30^ An example of a fine-tuned medical LLM is Google Med-PaLM 2, which has been evaluated through instruction tuning, including training on multiple-choice questions.^31^ Other approaches may also incorporate a reward model trained on human feedback (i.e., RLHF).^32^ Lehman et al. give additional examples of adapting LLMs for medical tasks.^33^ These may entail a general-purpose model injected with clinical data during pre-training or fine-tuning stages.^34^ Moreover, we observe a trend regarding the design of base models that includes an embedding layer for a set of prediction tasks,^35^ as well as a shift toward small language models.^36^ We welcome these developments, in principle, incorporating these future directions in our recommendations section. Specialized, medical LLMs are part of “rethinking” the approach to the design of general-purpose systems, leveraging the general capabilities of Large Generative AI with an “intended purpose” and use in mind.^37^

However, these specialized medical LLMs do not correspond to normative propositions required for the provider defining an intended use for risk management. Fine-tuning is a form of sophisticated task-optimization that can lock in model behavior while decreasing its complexity. By way of illustration, Med-PaLM has been evaluated by clinicians and non-clinicians for its general capabilities to engage with “medical exam questions.”^38^ Accordingly, it has been tested for its utility for medical question-answering using qualitative criteria, such as “scientific consensus,” truthfulness, or correct reasoning.^39^ Using this evaluative approach, the new “Med-PaLM-2” can introduce different data types and different modalities across medical disciplines.^40^ But as rightly put by Davenport, “[p]racticing medicine does not consist of answering medical questions … [and] the diagnosing (and possibly solving) of genuine clinical problems.”^41^ Specialized LLMs do not fulfill normative propositions about the context, the interactional implications, and evidence-based claims required, and are far from reflecting a robust account of an intended use. The upshot of this is that current design of these models inhibits narratives — for example, “multiple-choice accuracy” and “model capabilities” — that can actually produce *worse outcomes* for patients who do not readily “fit” the textbook style of questions.^42^

We further argue that fine-tuned and specialized LLMs will challenge providers to adopt a forward-walking logic for risk management. That is, task optimization clashes with the MDR requirement for articulating an intended use, as well as circumvents providers’ sequential and iterative framework.

### The Descriptive and Normative Lens of the Forward-Walking Logic

Medical LLMs pose unique risks for their evaluation under the MDR risk management framework. This can arise from dynamic interactions with the model via prompt engineering, trade-offs and limitations that can arise from examples of instruction tuning and RLHF. The following sections contend that the LLM’s general capabilities require providers to adopt more fine-grained and dynamic specifications. These specifications should include normative propositions for defining an intended use.

We illustrate the types of actions a developer could take for specialized LLMs, which are fine-tuned and adapted to a medical task. We identify three fine-tuning avenues in literature: utilizing medical literature and/or data, instruction tuning, and RLHF. We acknowledge the potential emergence of various approaches distinct from or intermediary to these methods, such as the role of Retrieval-Augmented Generation for task and output optimization.^43^ Nevertheless, our research provides a compelling case on why task optimization substantively contradicts current risk management reasoning. This allows for future work and alignment, considering future fine-tuning approaches.

**Figure 2:**
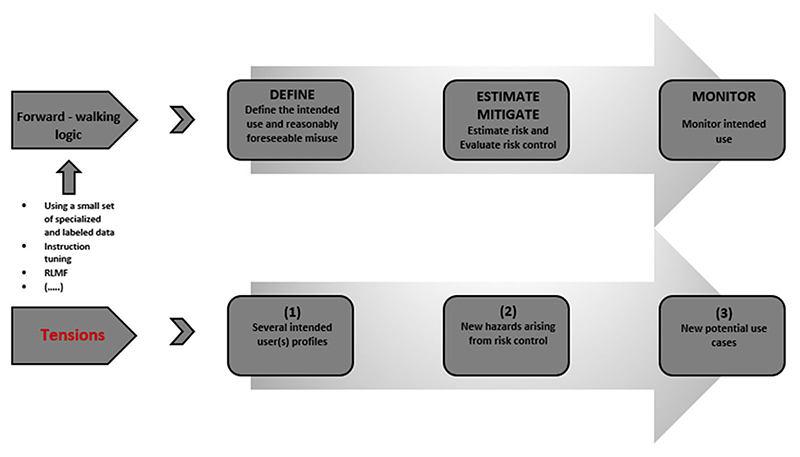
An outline — non-exhaustive enumeration — of the types of concerns that could arise based on the articulation of the intended purpose and use which in turn, require a set of different actions. We contend that these actions form three deviations from a risk policy. As a result, medical LLMs pose issues for complete specifications for risk management, while undermining the feedback loop on estimation, mitigation, and monitoring of risks.

We identify three areas of tensions corresponding to the three types of actions during the risk management cycle. In a nutshell, medical LLMs produce a set of deviations from the intended use and a risk policy during these risk management stages. These deviations are exemplified in Figure 2:

Imagine, for example, that our AI lung cancer detection software includes an interface which allows a healthcare professional, working in general practice, to upload medical records and chest X-rays. The healthcare professional can prompt the model in real-time for differential diagnosis during in-patient consultations. The tool is intended to be used for referrals on suspicion of lung cancer. It is intended to be used for patients who show common symptoms of lung cancer and are within an age group of 40–85 years of age. It is not intended for emergency care, nor for replacing clinical decision-making on follow-up treatments, such as patient referral to a computed tomography (CT) scan.

#### Several Intended Use(r) Profiles

The first area of tension is that with medical LLMs it is difficult to establish an intended use and reasonably foreseeable misuse for risk management. This is because with medical LLMs, the instances to define intended uses are much more dynamic than with task-specific models.

By way of illustration, the healthcare professional may prompt the medical LLM to “examine this X-ray and write a report for the presence of suspicious lung cancer.” However, depending on the specific prompt, the model might give a different answer to a similar question^44^ and prompt.^45^ Moreover, and depending on the level of detail and specificity of the prompt,^46^ the model might introduce new intended uses. Another healthcare professional, working in general practice, might pose a similar question for the detection of different lung cancer but formulated in the following way: “Examine this X-ray to rule out the presence of a lung abscess based on the patient notes [inserted here].” Here, the model might distinguish between different indicators, differentiating between lung cancer, a lung abscess, and empyema,^47^ and at different stages of severity. What follows is that the system now includes an intended use the detection of lung cancer regarding “patients suffering from any kind of lung disease.” Depending on the healthcare professional’s prompt the model might be incompatible with the original intended use.

Another issue is confirmation bias. In both illustrations above, the healthcare professional already has a suspicion that the patient might suffer from lung cancer or a lung abscess. This situation is not atypical, as a general practitioner is required to conduct physical examinations of the patient and gather information on their general health and symptoms before taking the X-ray. Nevertheless, LLMs show impressive capabilities to condense a vast amount of information and return the most probable response based on the specific prompt.^48^ Due to the model’s variability to introduce different intended uses, this may introduce additional concerns for the provider to predict reasonably foreseeable misuses. In other words, if the healthcare professional has omitted certain information in the patient notes, then the medical LLM will “insist” on certain patterns flowing from the prompt.^49^

The examples illuminate that the intended use and reasonably foreseeable misuse are not really the starting point for risk management. Rather, the dynamic interaction with intended users shapes the AI lung cancer detection software’s intended use and reasonably foreseeable misuse. The required specifications depend on the model’s output being shaped differently, depending on the intended user’s interactions and prompts. Following this reasoning, medical LLMs require more dynamic specifications for the intended use.

#### New Hazards Arising from Risk Control

We identify another tension, which is, if we were to insist on a specific intended use, that would produce incomplete specifications for risk estimation and mitigation from the outset. Incomplete specifications can give rise to the over- and underestimation of hazards during the system’s lifecycle. Incomplete risk estimates may produce trade-offs in risk evaluation and mitigation.

Now, imagine that providers introduce RLHF and instruction tuning to improve the quality and factual consistency of the output. It reveals that the model produces “hallucinations,” which means that it fabricates some responses. This occurs when the healthcare professional inserts the patient notes and the model then returns a diagnosis using a description of patient symptoms that are not present in these records.^50^ The human evaluators note that the system would fabricate patient symptoms indicating the presence of pneumonia and return (mis)diagnosis on empyema. These instances of the model producing “factually incorrect output” and/or “fabricated responses” are usually evaluated based on the model’s general capability to incorporate “clinical knowledge”^51^ and/or “clinical reasoning” in its output.^52^

The provider now wants to evaluate the device’s risks to produce factually incorrect output and fabricate responses. However, risks will have to be estimated based on the system’s general and medical question-answering capabilities *within* the device’s intended use. In our example, this would be difficult as we see that the system may fabricate information regarding a broad range of lung diseases such as lung cancer, lung abscess, pneumonia, and empyema.

Using a forward-walking scheme, providers would treat *all* these risks as “software failures” or a “novel hazard.”^53^ This means an estimation of the risks based on the “risks of the severity of harm alone,” given that incorrect and/or fabricated responses are “novel hazards” and “are difficult to estimate” based on the probability of harm.^54^ The upshot of this is that providers must rank these errors for their severity within the model’s broad spectrum and medical question-answering skills.

By way of illustration, estimating these hallucinations as an inherent feature of the system^55^ could distort overall risk estimation for measuring the severity of risks with an adequate level of “specificity.”^56^ On the other hand, treating hallucinations as the “worst-case severity of harm”^57^ would undermine risk management in a different way. Here, the provider would likely overestimate the risks, with the “worst-case severity of harm”^58^ leading to the worst outcomes even with small probability. In one situation we might have an overall risk assessment where hallucinations would produce the worst harm, making the system overall unreliable. In a different context, we might underestimate the risks, because we are ranking the inherent risks with low severity, while accepting the “risks of unpredictable reasoning hallucinations.”^59^ These are the two options introducing their own trade-offs based on incomplete specifications during risk estimation.

Nevertheless, risk estimation and evaluation will likely be “qualitative,” using RLHF and instruction tuning as examples of how to prevent *certain* errors from occurring.^60^ As noted by Elah, risk control measures can limit the severity of harm in cases where risks of harm cannot be estimated.^61^ However, the type of bench-marking for medical LLMs produces two problems for risk control. First, the model’s output for differential diagnosis is rarely “descriptive,” being a source of agreement and disagreement between annotators and clinicians.^62^ In this regard, benchmarking the model and its general capabilities against qualitative metrics, such as “comprehensibility,”^63^ would require the evaluation for an infinite amount of user experiences.^64^ Second, RLHF and instruction tuning can only optimize certain “patterns” in model reasoning and knowledge retrieval.^65^ This can include examples of RLHF used to “penalize obviously untrue statements,”^66^ and instruction tuning to improve accuracy on medical question-answering.^67^

Referring back to our example, the success of RLHF and instruction tuning would be rather limited. Human evaluators can certainly penalize instances where the system provides misinformation. For instance, Omiye et al. discuss that LLMs provide “indicators about kidney function and lung capacity … built on incorrect, racist assumptions.”^68^ Additionally, more examples of empyema can improve model reasoning slightly. However, our AI cancer detection software invents information subtly,^69^ as the indicators for pneumonia and empyema are not wrong per se. Rather, the patient might not suffer from pneumonia in the first place.^70^ Hence, the provider could not decrease the risks associated with the system hallucinating certain responses regarding lung cancer and empyema. Rather, the provider further optimized the system for additional tasks, while maintaining the risks of the model hallucinating in a static manner.

Hence, none of these measures decreases the risks in the design and use in a balanced way. On the contrary, human evaluation can correct some problematic model outputs, particularly on the relationship between lung cancer and common patient symptoms, while the model can still fabricate descriptors on lung cancer. Moreover, expert evaluation can introduce other biases into the model due to the “subjectivity of expertise,” for instance.^71^ These findings exacerbate the provider’s evaluation of the device’s *overall* risk and confidence levels *after* risk control. There is no balanced way for providers to demonstrate that risks — from the design to user specification errors — have been limited “as far as possible”^72^ and to an acceptable degree for our AI lung cancer detection tool.

These two types of trade-offs — using incomplete risk estimates and inconsistent risk control — produce new hazards during the system’s lifecycle. As noted by Bowan, techniques of “steering model behaviour” cannot ensure that the model will “behave appropriately in every *plausible* situation it faces in deployment” (emphasis added).^73^ Weighing risks for their severity will be increasingly difficult, due to the variability of risks across a broad spectrum based on the model’s capabilities. This in turn also affects the effectiveness of risk control and evaluation using task optimization for a model’s knowledge-retrieval and/or reasoning abilities. As a result, new risks and hazards are likely to arise inconsistently during the system’s lifecycle. This may indeed entail new errors — providing unreliable outputs — including suboptimal recommendation, incorrect information,^74^ and/or harmful advice.^75^

#### New Potential Use Cases

We identify a third tension in why medical LLMs clash with the provider’s forward-walking logic of risk management. This tension entails the medical LLMs’ “autodidactic function”^76^ to expand on new use cases, such as identifying new sub-populations when applied to real-world data and without direct supervision.

As noted by Minssen et al, one challenge for medical device regulation is “to manage adaptive learning in LLMs.”^77^ Contrary to AI-based medical devices retraining “incrementally” on the basis of new data,^78^ LLMs are positioned to adapt their responses in “realtime” depending on the prompt and/or context.^79^ These aspects — increased adaptability and decrease of human intervention — in turn, pose issues for providers, who have to monitor important changes to the device’s performance and effectiveness^80^ during the system’s lifecycle.

Changes to device performance and effectiveness can include new hazards and/or new claims the provider did not validate *ex ante*.^81^ For example, our medical LLM may learn new instances of symptoms, indicators, and lung diseases. Moreover, LLMs exhibit “few-shot or zero-shot learning” where the model can “unintentionally gain knowledge from implicit tasks in its training corpus.”^82^ These instances may create new claims, as well as new instances of failure models. For instance, wrong predictions of lung cancer require the provider to reevaluate the performance of the model. For our medical LLM this means that providers need to have a system in place that allows for real-time monitoring of these failure modes and usage for it to ensure that risks are at an acceptable level.^83^

However, our medical LLM does not only produce “performance-related hazards” and “failure modes.”^84^ Many risks of medical LLMs, including hallucinations, are inherent and systemic in the model architecture. How would the provider monitor whether the system arrives at the right answer? And how do you measure that the healthcare professional poses the right questions to the model? As noted by Hill et al, the MDR’s “post-market surveillance [framework] focuses on device malfunctions and serious injuries or deaths rather than maintaining ongoing device performance.”^85^ With medical LLMs, the borders between a model’s continuous functionality and a malfunction are much more fluid than with task-specific models.

Another aspect of risk management is that model reevaluation is proportional to the system’s original intended use and risk profile. This means that if our model learns new conditions for the detection of lung cancer, the provider must identify whether model behavior fits boundary specifications, such as the acceptable performance thresholds.^86^ A reference point for the provider is the device’s intended use for the detection of lung cancer. Nevertheless, if the model learns new “claims, intended uses or use conditions to the device,” this might require a new conformity assessment.^87^ This *may* apply to instances where the model may gain new “knowledge” on lung cancer for an extended patient cohort or new health tasks on the detection of lung diseases.^88^ Finally, our findings pertaining to the articulation of the intended use and reasonably foreseeable misuse further amplify risks for the provider to predict and monitor potential misuses and off-label uses of our medical LLM.

With medical LLMs, new potential use cases transcend (un) intended uses, while producing new hazards and failure modes. This can undermine the monitoring of emerging risks and hazards, as well as the reevaluation of the system’s intended use. The situation would be one in which the provider is testing intended uses, considering the model’s “broad functionality.”^89^

#### The Normative Implications of the Forward-Walking Logic

To summarize our points above, we contend that medical LLMs — pre-trained models adapted to a medical task — produce different tensions for providers to conduct risk management under the MDR. These tensions have normative implications in how providers of specialized LLMs ensure patient safety under the MDR. Intuitively, if the provider cannot formulate, refine, and reevaluate an intended use, that will produce incomplete specifications for risk management throughout the system’s lifecycle. Different intended user interactions, a broad spectrum of different risks with varying severity, and new potential use cases could endanger patient safety in multiple ways. We noted risks of the overestimation and underestimation of hazards, as well as inconsistent monitoring of performance-related hazards over ongoing functionality.

The implications of these issues are far-reaching, shaping our understanding of AI innovations, their intended uses, and utility in medicine. Considering the general capabilities, the uncertainty of risks and even hype surrounding their potential uses, regulators and providers need to reason around risk management with caution. As noted by Harrer, there are often “no second opportunities to get things right after releasing AI technology prematurely or hastily in the healthtech sector: user and regulator trust are easy to lose and very hard to regain.”^90^ The question arises, “What does a good risk management system look like under the new premises?”

### Walking Backward to Ensure Risk Management

We argue that an effective risk management system needs to flow backward, starting from the provider exploring model capabilities to the set of actions requiring dynamic specifications about the system’s intended use. The “backward-walking” logic prompts providers to approach risk management in the following way: “What can this model do within the specific Natural Language Processing (NLP) task X (i.e., question-answering, named entity recognition, etc.) and which intended uses would arise from this finding?”

**Figure 3:**
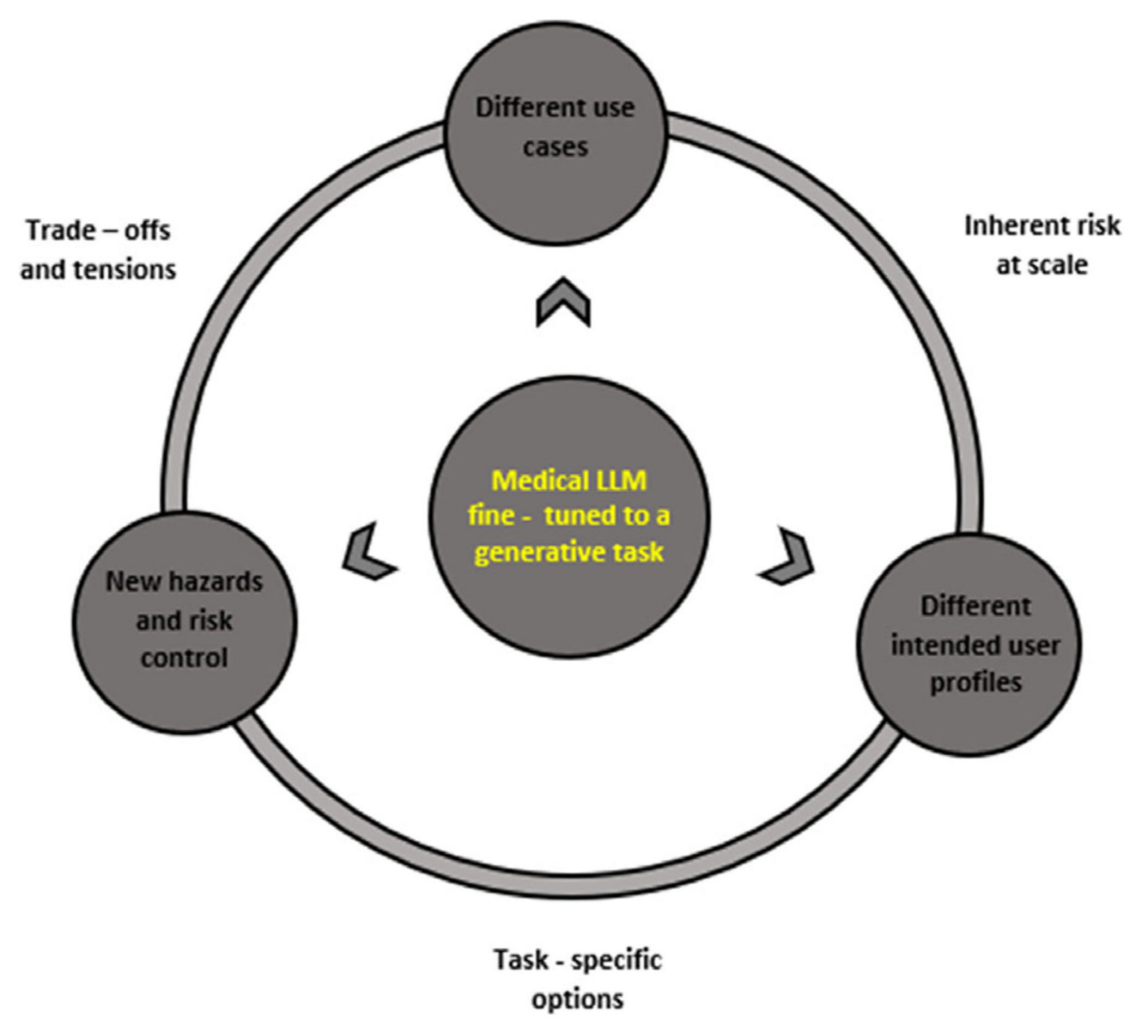
A revised logic of the MDR risk management frame-work using a “backward-walking” approach. Providers will use the model “general capabilities,” such as how well the medical LLM summarizes medical knowledge and engages in medical question-answering to define and reevaluate an intended use. The backward-waking logic works alongside the different deviations — intended users, new hazards, and potential use cases — to identify common and connecting factors. These are for providers to identify task-specific options and trade-offs, and to consider inherent risks at scale.

Crucially, our approach differs from the “forward-walking logic” as it allows providers to implement more dynamic, nuanced, and open-ended propositions about the model’s general capabilities to articulate an intended use. This is because providers are required to focus on what we call “sub-goals” for the articulation of the system’s intended use. Referring back to our initial example on the medical LLM for the detection of lung cancer, providers would be focusing on the set of actions adapting the model for a specific NLP or generative task. In the realm of computer science literature, a technique known as “backward-reasoning” or “backward-chaining” is an inference method for the model to provide evidence for a “goal” or “a hypothesis.”^91^ Rather than proceeding from initial data and facts in a “forward-chaining” manner, developers start with an initial “goal” and “sub-goals” driven by data to arrive at a conclusion that confirms a set of facts.^92^ Our understanding of a backward-walking approach similarly follows an implicit goal. Providers may have an idea about the LLM’s intended use for lung cancer detection to work with, but they need to work backward — from the types of actions to the types of concerns to risk management — to get there. This approach complements rather than replaces the current risk management approach in the MDR, focusing specifically on the “sub-goals” to define, estimate, mitigate and monitor of risks of specialized LLMs. Figure 3 introduces and simplifies this idea of backward-walking logic within the MDR risk management frame-work.

The backward-walking logic entails the consideration of subgoals — what the different intended user(s), risks of new hazards and different use cases are — and finding connections along a spectrum of three elements. These include the task-specific options, inherent risks at scale, and trade-offs.

#### Task-Specific Options

The first connecting factor is that providers must identify how dynamic human-AI interactions produce different use cases and intended users. A lot of research is directed toward advanced prompt engineering to evaluate the LLM’s clinical reasoning abilities. For example, Wei et al. propose a method for the model to break up its task into smaller reasoning steps.^93^ Another method would be for the provider to constrain the model responding to a specific scope and/or excluding certain out-of-scope (including harmful) responses to user prompts.^94^ Finally, Thirunavukarasu et al. advocate that the model output needs to display an “uncertainty indicator” for accuracy.^95^ These are indeed useful methods for the provider to define reasonably foreseeable misuses and test design specifications for their usability. In this regard, it is argued that prompt engineering is going to be an “essential skill” for the intended user, the healthcare professional, to understand the practical utility and limitations of the LLM for a *desired* task.^96^

For providers incorporating these methods and considerations into their definition of the intended use, clear demonstrations of the system’s safety and performance regarding an *actual* task are required. Providers evaluating the model through prompt engineering, RLHF, and instruction tuning must arrive at a set of task-specific options. These task-specific options could include what types of questions this model can be used for, how these questions need to be formulated, and what the scenarios are in which the use of this model solves an unmet clinical need. These are some ways that providers can test and refine the intended use from the beginning. Our breakdown of the intended use to task-specific options is intended to go further than the evaluation of the system’s medical question-answering capabilities, to look at the different interactional implications to test, refine and optimize the model’s output.

In this regard, we see the emergence of small language models, as well as domain-specific models to enhance the provider’s refinement of task-specific options. For example, Google recently developed a series of “health-specific embedding tools” using a domain-specific model and compressed in a general-purpose system.^97^

Nevertheless, this assessment also includes trade-offs. For instance, small language models and specialized, medical LLMs do not eliminate risks of the model hallucinating.^98^ Therefore, the provider needs to examine how different intended uses produce trade-offs for risk estimation and control.

#### Trade-Offs

The trade-offs in risk estimation and risk mitigation are important sub-goals for providers to judge the effectiveness of risk control. Hallucinations in (medical) LLMs illustrate an important area for providers to examine these trade-offs. There is some literature that intends to evaluate issues surrounding hallucinations in medical LLMs. Omiye et al. note that prompts that include “insufficient information” worsen model hallucinations.^99^ Hence, usability testing might give some insight into how and to what extent some risks of automation bias can be minimized through the user’s formulation of the prompt. It is important to note that the provider needs to document these trade-offs, which risks can be mitigated, and to what extent that depends on the formulation of the task and/or instructions.

Accordingly, this sub-goal needs to direct the provider to reevaluate the intended use. In our example of the medical LLM for detection of lung cancer, an open-ended question on the “common risk factors of lung cancer and lung abscess” may yield a wider array of responses on diverse topics, while producing a distinct set of hazards relevant to risk estimation and evaluation. RLHF cannot limit inherent risks but can provide insights on which *residual risks* might be tolerable for a set of actual tasks, questions, and inputs. Referring back to the importance of task-specific options, this assessment should demonstrate in what way risk control can eliminate certain risks, and how the residual risks are justified for which tasks.

#### Inherent Risks at Scale

It is also important for providers to consider the scale of change and iterations when LLMs operate on the ground. Providers need to consider the model’s “fickle” nature to “evolve rapidly” and produce unpredictable outputs.^100^ This may entail “real-time monitoring” of medical LLMs performance and safety.^101^ Gilbert et al. suggest a separate oversight layer, entailing the “automated real-time fact checking of model output.”^102^ Further, regulatory guidance needs to clarify how providers can predetermine model updates within the confines of the device’s intended use.^103^ This would entail new boundary specifications, considering qualitative evaluations from RLHF, and instruction tuning. In particular, boundary specifications need to define different degrees of “systematic” misuse for the provider to identify and monitor the “correct intended purpose” and use.^104^

As a result, the backward-walking logic clearly has an added benefit to ensure patient safety of medical LLMs. This is because it is a framework that encourages providers to engage in more hypothesis-driven, exploratory work to articulate an intended use. In doing so, the backward-walking logic does not change the risk management framework requiring definition, estimation, mitigation, and monitoring of risks. Nevertheless, it encourages providers to reason around the risks of specialized LLMs in a balanced way. Using the connecting factors, the provider can define the device’s intended use and navigate risk management.

#### Limitations

There are clear limitations of this research. The backward-walking logic is a system for providers complying with EU sectoral legislation when their model is “developed for, or adapted, modified or directed toward specifically medical purposes.”^105^ For it to be effective, however, requires consolidation of the entire value chain of LLM development. For example, many open-source LLMs would count as Software of Unknown Provenance (SOUP). SOUP in a medical device is software, or a software item, developed by a third party and would require the provider of the medical LLM to analyze these software items within ISO IEC 62304.^106^ Hence, new requirements for technical documentation and transparency upstream are needed for providers of medical LLMs to test the system and software components’ overall performance and estimate risks. The backward-walking logic only intends to inform the responsibilities of the manufacturer of the medical device conducting risk management. An aspect of future research is understanding the backward-walking logic directing the actions of general-purpose system providers and SOUP manufacturers upstream.

## Conclusion

The MDR’s risk management framework provides a process for manufacturers of medical devices to reason around the device’s intended purpose and use. It clearly outlines a sequential process, starting from the manufacturer’s definition of the intended use and reasonably foreseeable misuse, and moving forward to risk control and reevaluating the device’s intended use. The MDR’s forward-walking model is designed to assume that a system performs a specific task, while manufacturers define risks in relation to that specific task. LLMs break that model to the extent that risk management can produce incomplete definitions about a system’s medical purpose and intended use and additional hazards arising from risk control and offer no system to monitor task-performance. Therefore, we argue that the forward-walking logic needs to be changed, while maintaining the discrete aims of risk management to follow a specific process. We refine the MDR’s risk management process in a way that guides providers to explore different tensions. These include how different intended user profiles, new hazards and new potential use cases reinforce a definition of the device’s intended purpose and use. Rather than beginning with an intended purpose and use, application developers will need to examine the system’s general capabilities to articulate different intended uses and corresponding risk. We describe this new approach as backward-walking logic, as it prompts application developers to reflect on the goals of risk management differently. In this respect, providers need to reassure themselves what the model can do within the specific NLP task X (i.e., question-answering, named entity recognition…) and which intended uses would arise from this finding.

Our approach appreciates that providers need to explore connecting factors between the intended uses and risk profiles. This requires a breakdown of task-specific options for evaluating the LLM, including assessing model safety and effectiveness in different use cases and with various intended users. Furthermore, the backward-walking logic supports providers to document trade-offs and tensions of fine-tuning and effectiveness of risk control. Finally, specific risks, including the risks of hallucinations, require dynamic and open-ended definitions of a risk profile to enable real-time monitoring.
